# Identification of key factors causing ketosis in dairy cows with low feed intake

**DOI:** 10.1080/10495398.2025.2487089

**Published:** 2025-04-04

**Authors:** Xue Feng, Qi Feng, Sayed Haidar Abbas Raza, Fen Li, Yun Ma

**Affiliations:** ^a^ Key Laboratory of Ruminant Molecular and Cellular Breeding of Ningxia Hui Autonomous Region, College of Animal Science and Technology, Ningxia University, Yinchuan, China; ^b^ School of Computer and Information Engineering, Henan University, Kaifeng, China; ^c^ Research Center for Machining and Safety of Livestock and Poultry Products, South China Agricultural University, Guangzhou, China

**Keywords:** Ketosis, RFI, feed intake, dairy cows, WGCNA

## Abstract

Ketosis is a common metabolic disease in high-yield dairy cows. Key genes affecting ketosis need to be further explored by new methods. The gene expression profiling and clinical data of GSE92398, GSE104079, and GSE4304 were obtained from the gene expression omnibus (GEO) database. Core modules and genes associated with RFI (residual feed intake) and ADF (alternate day fasting) were identified by weighted gene co-expression network analysis (WGCNA). Subsequently, the key genes related to ketosis and RFI were determined by protein-protein interaction (PPI) networks, ROC curves, functional enrichment, and differential expression analysis, respectively. The results showed that the genes of ACACA, ELOVL6 and XPO7 could be used as regulators of ketosis induced by low feed intake in dairy cows. At the same time, three genes (HRFI, STAT3 and IFNAR1) were retained as additional RFI biomarkers that could be considered. We identified three key factors as candidate genes and biomarkers of ketosis and RFI, respectively. These factors may provide a theoretical basis for targeted therapy of ketosis in dairy cows.

## Introduction

Residual Feed Intake (RFI) measures whether the animal’s actual feed intake is above or below the predicted by published feeding standards [1,2] and is defined as the difference between the actual and predicted dry matter intake (DMI) [3]. Variation in RFI may be related to five major physiological processes, which include intake, digestion, metabolism (anabolism and catabolism associated with and including variation in body composition), physical activity, and thermoregulation [4]. Among them, Herd et al’s RFI divergent selection of cattle estimated [2] that heat production from protein turnover, tissue metabolic, and stress accounts for 37% of the variation in RFI. This indicates that the variation of RFI is closely related to metabolism. Indeed, it is. Alexand et al. [3] found that lipid metabolism in the liver of beef cattle with low feed efficiency (LFE) was altered. However, RFI, as a widely accepted measure, is used more to measure animal feed efficiency (FE) [3,5–8]. The definition of feed efficiency in dairy cows must take into account the catabolism and anabolism of body reserves, so selecting dairy cows that covert feed into milk is the primary objective. A number of methods have been developed and used to select the dairy cows with the highest FE. Among them, low RFI animals have more FE (HFE, high feed efficiency) [9] and less food intake (DMI) than high RFI animals.

The effect of RFI on dairy cows should be take into account at this stage, mainly because cows with lower RFI seem to be more efficient but also have a more severe negative energy balance (NEB) [10–12], which can lead to health problems and affect fertility in dairy cows [12]. Previous studies have extensively demonstrated that NEB marker [13], such as non-esterified fatty acids (NEFA) and β-hydroxybutyric acid (BHBA) in serum, are associated with the incidence of clinical postpartum disorders such as ketosis. Ketosis is one of the most important metabolic disorders during transition period, which is cause by a serious imbalance between energy demand and energy intake. Although ketosis is curable and rarely causes the death of dairy cows, is can reduce the milk yield [14] and reproductive performance [15] of dairy cows, including reproductive system disease and endocrine disorders [16]. Thus increased the cost of treatment and the risk of early culling, resulting in serious economic losses to the livestock industry. Therefore, the identification of the genes related to ketosis can provide the relevant biomarkers for the early diagnosis and prevention of ketosis in dairy cows, thus proving theoretical support for finding effective therapeutic strategies for ketosis in dairy cows.

The sharing of omics data has pushed biological research into a fast lane. Free and unconditional use of data is indicated in existing data guidelines published in the public domain, which will further accelerate the development of systematic biological research. Moreover, systems biology research driven by multi-omics will also become a new paradigm of life science research. High-throughput transcriptomics and microarray technology are wide used to quantify gene expression in specific cell type or tissues and to gain insight into gene function associated with a particular trait. In recent years, there have been studies to identify candidate genes that influence metabolic disease (such as ketosis), milk yield and so on through RNA-seq and microarray analysis of the liver in dairy cows [17,18]. As a complex network system of eukaryotes, its life activity is mainly carried out by gene and gene interaction between the function. However, WGCNA is a biological approach that can be used to correlate the expression of genes in sample with phenotype [19–21], by which relevant networks can be constructed to identify candidate biomarkers or therapeutic targets [22]. At present, it is widely used in various biological background [12,23–26]. At present, the research about the ketosis is also increasing gradually [27,28]. If gene associated with ketosis can be found to be associated with low RFI using public datasets, this seem to be a novel approach for identifying new candidate genes associated with ketosis.

We queried the data set for the keyword “DMI/RFI” and “ketosis” in the GEO database and identified a set of data set to study the high and low RFI of dairy cows (GSE92398, Holstein). At the same time, we also noticed another set of data (GSE104079), which is the mice fasting treatment. Because fasting is the most potent physiological stimulus for ketosis, Marosi et al. designed this study to determine the effect of intermittent fasting during endurance training on performance and to elucidate potential cellular and molecular mechanisms. The material handling of this data set is consistent with our research objectives. Therefore, we hypothesized that some key genes may be significantly associated with the development of ketosis, whether in fasting-induced ketosis in mice or low-DMI-induced ketosis in dairy cows. The last dataset, GSE4304 (Holstein) [17], provides a set of differentially expressed genes between ketosis cows and healthy cows. Therefore, base on “RFI”, “Fasting” and “ketosis”, these three public datasets are the main data sources for screening new candidate genes in this study. In this study, WGCNA was used to construct gene co-expression network to analyzed module genes significantly related to low RFI (High FE) in dairy cows. The overlapping genes between these module genes and the differentially expressed genes in ketosis are regarded as new candidate genes for ketosis caused by low feed intake (Low RFI) in dairy cows.

## Materials and methods

### Data collection and preprocessing

**Figure 1.**
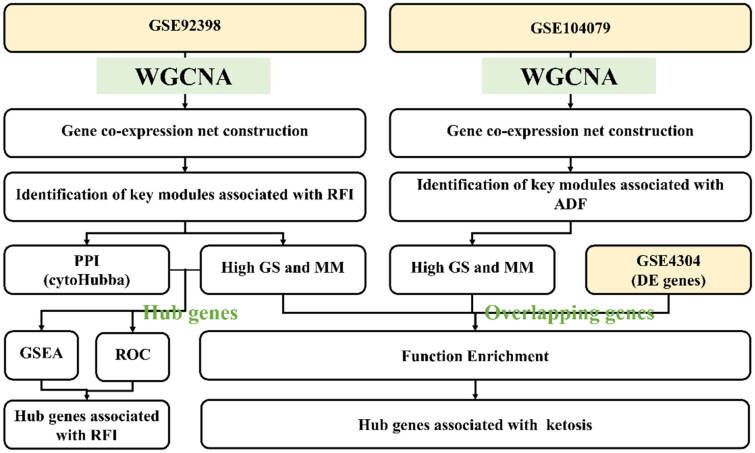
Flow chart of experiment design.

The transcriptome data set used in this study was downloaded from the Gene Expression Omnibus (GEO) database (https://www.ncbi.nlm.nih.gov/geoprofiles). The data set number, GSE92398, provided a gene expression profiling of 18 samples containing two sets of RFI (LRFI and HRFI) data for dairy cows. The data set, GSE104079, provided a gene expression profiling of 58 samples containing both sets of mice ADFs (SADF and EXADF). The last dataset, GSE4304 [17], provides a set of differentially expressed genes between ketosis cows and healthy cows. Both cow datasets were sequenced from early lactating Holstein liver tissue (GSE92398 and GSE4304). Similarly, the mouse data set also involved liver tissue (GSE104079). Detailed experimental and phenotypic information for the three datasets is included in Supplementary Table 1. The flow chart of this study is shown in Figure 1.

Transcriptomic data from the GSE92398 dataset, comprising 18 samples of liver tissue from 9 early lactating Holstein cows, were analyzed. In the same feeding management conditions, the distribution of different diets (High concentrate and Low concentrate). Dietary composition can be referred to previous studies [9]. Each cow underwent two dietary phases (high-concentrate [HC] and low-concentrate [Control, C]) in a crossover design. Simply put, 5 cows initially fed a high-concentrate diet (HC) and 4 cows a low-concentrate diet (LC). After 14 days, the diets were swapped (HC→LC and LC→HC) for an additional 14 days, with liver biopsies collected at the end of each phase. The liver tissue from the biopsy was used for subsequent RNA sequencing. After removing one outlier sample (GSM2429026) during quality control, 17 samples (8 HRFI and 9 LRFI, Supplementary Figure 1) were retained for analysis. Among them, LRFI stands for low RFI, HRFI stands for High RFI.

The processing of the GSE4304 dataset is similar. Fourteen early lactating Holstein cows were retrieved from data set GSE4304. Under the same feeding management conditions, different feeds (Feed diet and 50% Feed diet) were distributed. Similarly, the dietary structure can be referred to previous studies [17]. Fourteen post-natal Holstein cows (healthy cows) were randomly assigned to the healthy control group (n = 7) and the ketosis induction group (n = 7) at 4 days postnatal. Ketosis was induced by feed restriction until clinical signs of ketosis appeared or until 14 days postpartum. Then resume to take food at will, undertake treatment when necessary. Similarly, liver biopsies taken from liver tissue samples found more than 9,000 sequences representing microarray expression in the liver. The mouse data set (GSE104079) serves as a reference for different species and is not described in detail here, as shown in Supplementary Table 1 and previous descriptions [29]. Among them, a sedentary group of mice on alternate-day fasting (ADF) was represented by SADF, and a group that ran every day on a treadmill while on ADF during a 4-week study period were represented by EXADF.

To obtain the consistent expression matrix, we used the pipeline as follows to quantify the gene expression of the datasets (GSE92398). Sequencing quality was checked using FASTQC (version 0.11.3), and adapters were removed using cutadapt (version 1.6) [30], and based on the quality control report, the reads were not further pre-processed. The ultrafast universal RNA-Seq Aligner STAR (version 2.3.0) [31] aligns reads with genome-assembled Bos taurus UMD3.1.

### Construction of co-expression network

**Figure 2.**
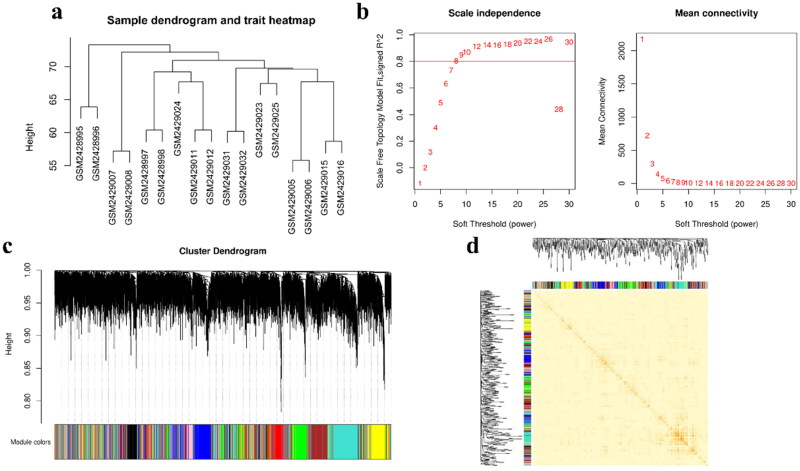
Established of gene co-expression network. (a) Cluster analysis diagram of sample. (b) The scaling-free fitting indices (left) and the mean connectivity (right) of various soft threshold powers are analyzed. (c) Cluster analysis diagram of co-expression module. (d) Heatmap drawing of co-expression module.

The gene co-expression network constructed in this study utilizes the WGCNA package in R (version 4.1.0) [20] (GSE92398 and GSE104079, the following analyses are performed simultaneously in both datasets, except where otherwise indicated.). The detailed operation process has been embodied in the previous [23,24]. Simply put, the top 12,000 genes were selected based on median absolute deviation (MAD) to reduce noise. Expression matrices were log2-transformed and normalized using the normalize.quantiles function in R. The pickSoftThreshold function in the WGCNA package was used to determine the optimal soft threshold power (β). We evaluated scale-free topology fit (R^2^ ≥ 0.8) and mean connectivity for β values ranging from 1 to 20. A β = 6 was selected for GSE92398 (cow RFI dataset) and β = 8 for GSE104079 (mouse fasting dataset) to ensure scale-free network properties (Figure 2b, Supplementary Figure 2b). A signed adjacency matrix was converted into a topological overlap matrix (TOM) to minimize spurious correlations. Hierarchical clustering with dynamic tree cutting (minimum module size = 30, merge threshold = 0.25) partitioned genes into co-expression modules.

### Identification of modules significantly associated with RFI and ADF

The module eigengene (ME) is considered to represent the gene expression profiling in a module, mainly because it is considered as the first principal component of a given module. To identify module genes associated with RFI (LRFI/HRFI) and ADF (SADF/EXADF), the correlation between MES and phenotypes was evaluated using Pearson’ correlation validation with *P* < 0.05 as the cutoff, and the most significantly modules were further analyzed as key modules. Simply put, Pearson is a value between −1 and 1 that describes the tendency of two linear sets of data to move together. In mathematical terms, the Pearson correlation coefficient is equal to the covariance of the two variables divided by the standard deviation of the two variables (MES and RFI/ADF). As follows.

$$\begin{array}{c} r=\frac{N\sum x_{i}y_{i}-\sum x_{i}\sum y_{i}}{\sqrt{N\sum x_{i}^{2}-{\left(\sum x_{i}\right)}^{2}}\sqrt{N\sum y_{i}^{2}-{\left(\sum y_{i}\right)}^{2}}},\\ \text{Correl}\left(X,Y\right)=\frac{N\sum \left(x-\bar{x}\right)\left(y-\bar{y}\right)}{\sqrt{\sum {\left(x-\bar{x}\right)}^{2}\left(y-\bar{y}\right)}}; \end{array}$$

### Identification of hub genes

The first principal component (ME) of each module was computed to represent overall expression patterns. Pearson correlation analysis between MEs and traits (RFI, ADF) identified modules significantly associated with phenotypes (*P* < 0.05). GS (Gene significance) and MM (module membership) were calculated. Genes with GS > 0.4 and MM > 0.8 were prioritized as potential hub genes [20,32]. Among them, GS defines the correlation between gene expression and a specific phenotype. MM are defined as associations between gene expression and each ME [20]. The equation is shown below. ${\text{GS}}_{m}=|\text{cor}(x_{m},T)|$; ${\text{MM}}_{m}=|\text{cor}(x_{m},E^{n})|$.

Furthermore, the R package (ggplot2, stringr, enrichplot and clusterProfiler) is used to enrich the function of these potential genes and display them visually. Protein-protein Interaction Networks (PPI) are analyzed by STRING (https://cn.string-db.org/). Hub genes were submitted to the STRING database to generate interaction networks. Cytoscape (v3.8.0) was used to visualize and analyze the network. Four centrality algorithms (Betweenness, Degree, EPC, and MNC) were applied to rank nodes. Overlapping top-ranked genes across all algorithms were defined as key regulators. Use an online website (http://sangerbox.com) to draw Venn diagram.

### Functional enrichment analysis

The functional enrichment only analyzes the data set GSE92398. As mentioned in previous research [24], the Gene Ontology (GO, “org.Bt.eg.db”, GSE92398), Kyoto Encyclopedia of genes and genomes (KEGG, “bta”, GSE92398) pathway and Single-gene GSEA (Gene set enrichment analyses) were performed using the “Clusterprofiler” package (*p*.adjust <0.05) in R soft (R version 4.2.2).

### Differential expression validation and efficacy evaluation of hub genes

The potential value of hub genes was further verified by measuring the differential expression patterns of candidate hub genes in HRFI and LRFI and their ability to distinguish high RFI and low RFI levels. Among them, ROC curve plotting showed an assessment of the ability to have both high and low RFI levels, which was mainly reflected in the area under curve (AUC) of ROC calculated from the “pROC” package in R.

## Results

### Construction of co-expression network

The dataset (GSE92398) with high and low RFI was selected for WGCNA analysis, aiming at mining hub genes associated with ketosis induced by RFI. Cluster analysis of the 17 samples showed no additional outliers, which can be further analyzed (Figure 2a). When constructing co-expression networks, the soft threshold is determined by choosing a correlation coefficient threshold of 0.8 (Figure 2b). Nine co-expression modules were identified by WGCNA analysis (Supplementary Table 2). Of these, the module containing the largest number of genes was the turquoise module (1,544 genes; 15.5%), followed by the blue modules (1,319 genes; 13.3%) and the brown module (1,161 genes; 11.7%) (Figure 2c). And network heatmap analysis showed that genes within each module tended to exhibit higher connectivity, but each module was independent of the others (Figure 2d).

Similarly, considering that fasting is the most effective physiological stimulus for ketosis, we also analyzed the dataset (GSE104079) of fasting stimuli for WGCNA. To explore some key genes that may be involved in the development of ketosis, these genes are present not only in ketosis induced by fasting in mice but also by low DMI in cows (Supplementary Figure 2). Similarly, the dataset GSE104079 was clustered for further analysis (Supplementary Figure 2a). A correlation coefficient threshold of 0.8 was used to determine the soft threshold (Supplementary Figure 2b), and the analysis identified 5 co-expression modules (Supplementary Figure 2c). Genes within each module also showed higher connectivity (Supplementary Figure 2d).

### Analysis of modules correlated with RFI

**Figure 3.**
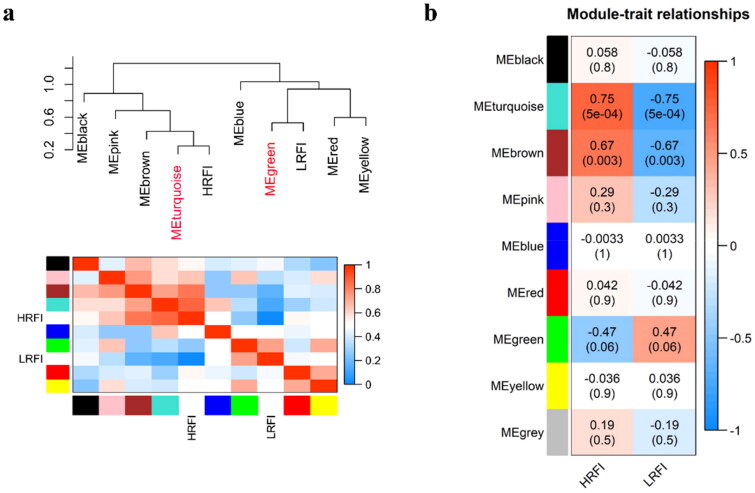
Co-expression analysis of modules and traits. (a) Cluster analysis diagram between modules and traits. (b) Correlation heatmap between modules and traits. The positive and negative numbers represent the correlation coefficient, the values in parentheses represent the significance *P*-value.

**Figure 4.**
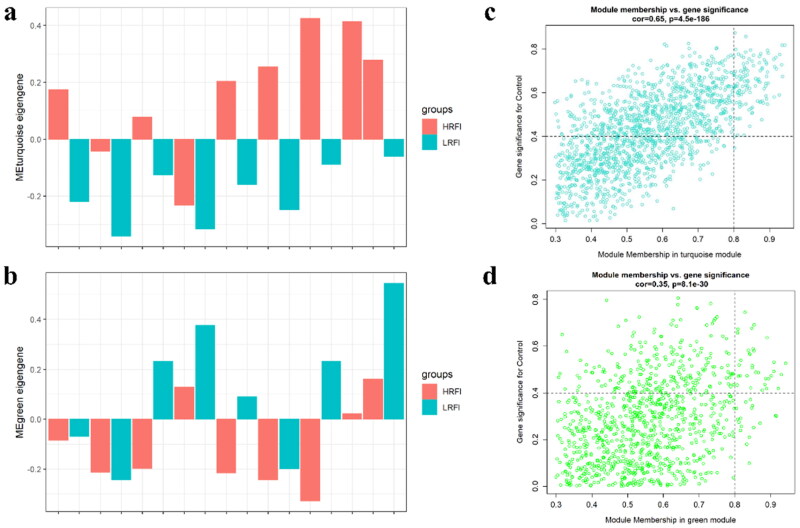
Description of ME and genes distribution in different modules. (a) Turquoise modules associated with HRFI. (b) Green modules associated with LRFI. (c) The GS and MM value in the turquoise module. (d) The GS and MM value in the green module. The turquoise and green dots represent genes number in the corresponding module.

**Figure 5.**
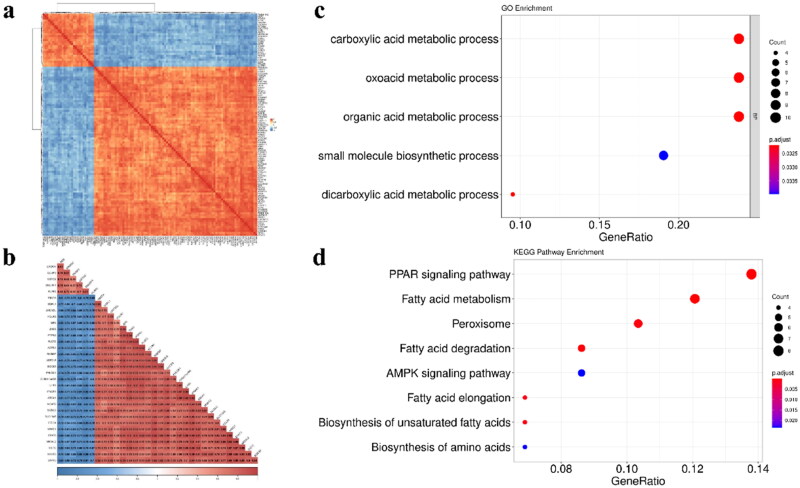
Correlation analysis and functional enrichment analysis of potential hub genes. (a) Heatmap analysis of the potential hub genes in turquoise modules. (b) The correlation relationship of the potential hub genes with high GS and MM value (GS > 0.4, MM > 0.8) in green modules. (c) GO function enrichment analysis of the potential hub genes. (d) KEGG function enrichment analysis of the potential hub genes.

Two modules (Turquoise module and green module) were found to be correlated (negative and positive) with traits by correlation analysis (Figure 3a and b). It revealed that the samples of RFI (HRFI/LRFI) have higher ME values in the module (Turquoise/Green) (Figure 4a and b). We identified genes of high values in 2 modules (GS > 0.4, MM > 0.8) and found that these genes had high correlations with each other, suggesting that these genes are potential hub genes (Figure 4c and d and Figure 5a and b). GO functional enrichment analysis revealed that these genes were mainly enriched in “Carboxylic acid metabolic process”, “Oxoacid metabolic process”, “Organic acid metabolic process”, “Small molecule biosynthetic process” and “dicarboxylic acid metabolic process” (Figure 5c, Supplementary Table 3). Similarly, KEGG was found to be enriched in “PPAR signaling pathway”, “Fatty acid metabolism”, “Peroxisome”, “Fatty acid degradation”, “AMPK signaling pathway”, “Fatty acid elongation”, “Biosynthesis of unsaturated fatty acids” and “Biosynthesis of amino acid” (Figure 5d, Supplementary Table 3). According to the enrichment results, we can find that these genes are involved in the disorder of glucose and lipid metabolism in the body.

As shown above, the same analysis was performed again in the dataset (GSE104079) and identified 269 potential hub genes with association with ketosis (Supplementary Figure 3). Two modules (Brown and Yellow) were found to be associated with traits (EXADF, negative and positive) by correlation analysis (Supplementary Figure 3a and b). 269 potentially high-value genes associated with ketosis (GS > 0.4, MM > 0.8) were identified in 2 modules (Supplementary Figure 3c and d).

### Identification of hub genes correlated with RFI

**Figure 6.**
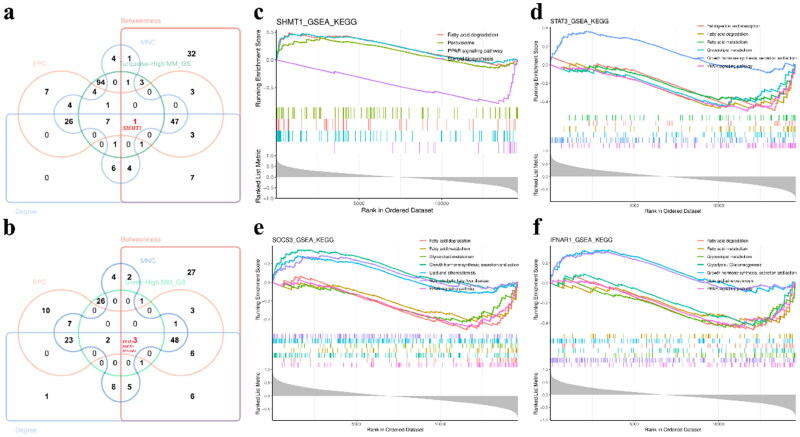
Identification of key hub genes and GSEA enrichment analysis. (a) Intersection of potential genes (1,544) and hub genes (103*4) identified based on four algorithms (Betweenness, Degree, EPC and MNC) in turquoise and green modules. (b) Intersection of potential genes (987) and hub genes (103*4) identified based on four algorithms (Betweenness, Degree, EPC and MNC) in turquoise and green modules. (c-f) GSEA analysis of key hub genes associated with RFI. The horizontal axis represents the ranked genes list in related to the target gene. The numbers above the vertical coordinate indicate the enrichment score, and the number below the vertical axis indicate the correlation coefficient with the target genes. Each color represents the pathway in which the target gene participation.

We identified a total of 146 key potential hub genes in turquoise (113) and green (33) modules (GS > 0.4&MM > 0.8). Further, all genes of the 2 modules were sent to STRING to construct PPI (Protein-Protein Interaction) network, and top 103 genes were identified by using Betweenness, Degree, EPC and MNC algorithms, respectively (Supplementary Figure 4). Finally, it was found that SHMT1, STA3, SOCS3 and IFNAR1 were the key hub genes identified by intersection of four algorithms with potential hub gene sets (Figure 6a and b). GSEA enrichment analysis of key genes, it was found to be mainly enriched in "Fatty acid degradation", "Peroxisome", "PPAR signaling pathway", "Steroid biosynthesis", "Fat digestion and absorption", "Fatty acid metabolism", "Glycerolipid metabolism", "Growth hormone synthesis, secretion and action", "Lipid and atherosclerosis", "Non-alcoholic Fatty liver disease", "Glycolysis hormone synthesis, secretion and action" (Figure 6c–f).

**Figure 7.**
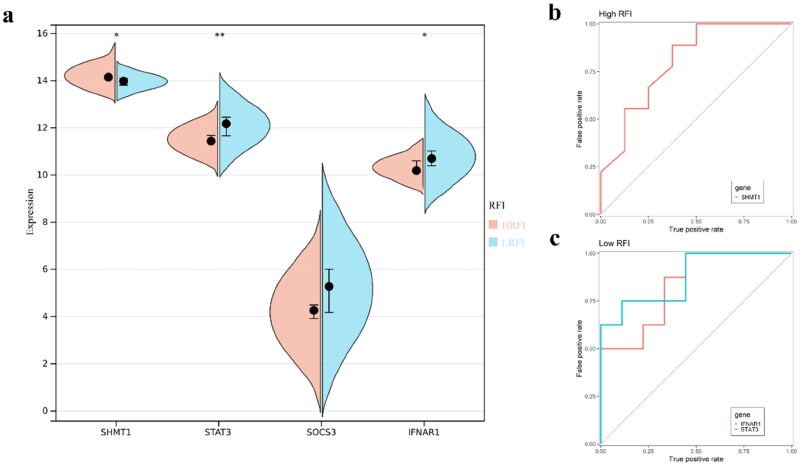
Differential expression validation and efficacy evaluation of key genes. (a) Differential expression analysis of key genes. (b) ROC curve analysis of key genes for the ability to distinguish between HRFI and LRFI. The number of colored lines represent the number of genes. The area of the ROC curve determines the ability of the target genes to distinguish.

The expression of SHMT1 was significantly up-regulated in HRFI, STAT3, SOCS3 and IFNAR1 were more high expression lever in LRFI (Figure 7a). Next ROC plots were drawn to determine the ability of the three genes to distinguish between HRFI and LRFI. It was found that SHMT1 could distinguish HRFI better than STAT3 and IFNAR1 (Figure 7b and c).

### Potential genes associated with ketosis were analyzed

**Figure 8.**
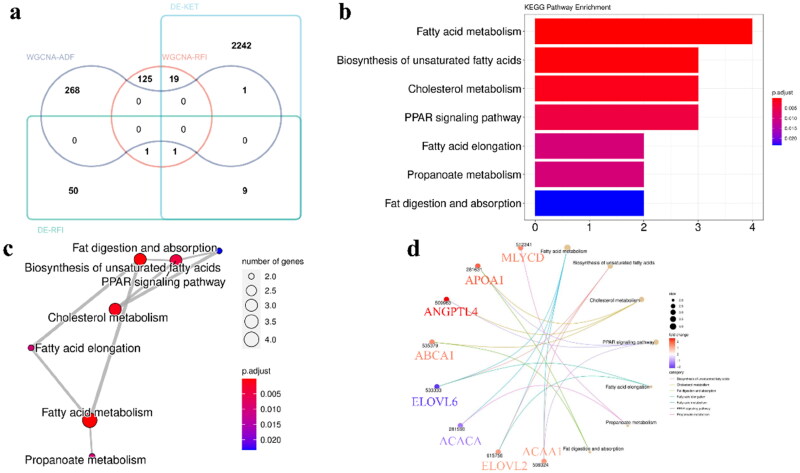
Identification of key genes related to ketosis and functional enrichment analysis of KEGG. (a) The key 31 genes related to ketosis were identified base on the gene sets of DE-ketosis (2,272 genes, |log2FC|>1 & *P* < 0.05), WGCNA-RFI (the hub gene of the turquoise module and the green module, 146 genes), DE-RFI (70 genes, *P* < 0.05) and WGCNA-ADF (the hub gene of the brown module and the yellow module, 269 genes). (b) KEGG function enrichment analysis of key genes. (c) Network diagram of the enrichment pathways. (d) a network diagram of genes and signaling pathways.

To better search for candidate genes associated with ketosis, we took overlapping analysis of key genes of DE-ketosis [17], WGCNA-RFI, DE-RFI [9] and WGCNA-ADF. Unfortunately, we did not find all the overlapping genes in the four plates. Therefore, we focused on all the overlapping genes and identified them as candidate genes (31 genes) (Figure 8a, Supplementary Table 2). Among them, DE-ketosis represents a gene set that was differentially expressed in ketosis cows compared with healthy cows, and DE-RFI represented gene set that was differentially expressed with HRFI and LRFI. The data on DE-ketosis and DE-RFI were derived from the dataset GSE4304 [17] and GSE92398 [9], respectively.

We performed KEGG functional enrichment analysis on 31 overlapping candidate genes, it was found that the main enrichment pathways were Fatty acid metabolism, Biosynthesis of unsaturated fatty acid, Cholesterol metabolism, PPAR signaling pathway, Fatty acid elongation, Propanoate metabolism, Fat digestion and absorption (Figure 8b–d).

**Figure 9.**
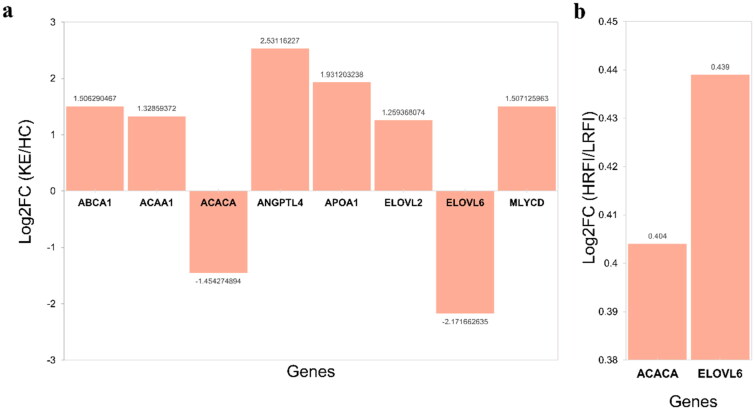
Differential expression analysis of key genes. (a) Differential expression analysis of key genes in ketosis and healthy dairy cows. (b) Differential expression analysis of key genes in HRFI and LRFI.

Further, we identified genes involved in these pathways as potential key genes for differential expression analysis. It was found that ACACA and ELOVL6 were differentially expressed in HRFI and LRFI, and also in healthy and ketotic cows, which could be considered as candidate genes for ketosis due to low food intake (Figure 9a and b).

## Discussion

**Figure 10.**
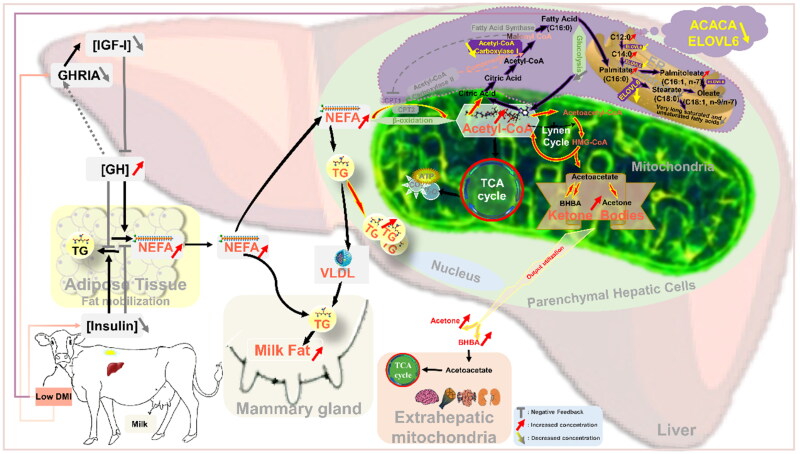
Ketosis in dairy cows caused by DMI results in changes of metabolic pathways in the body.

Cows with significantly lower DMI are at increased risk for a variety of metabolic diseases, often associated with NEB, such as ketosis. When the dairy cows DMI is reduced, and priority id given to the breast’s nutrient requirements [33,34], nutrient intake is not enough to meet the body’s energy need, leading to the body in negative energy balance (NEB). NEB initiates adipose tissue mobilization, causing triglyceride (TG) to be hydrolyzed into NEFA. High concentrations of fatty acids impair insulin signaling pathways and decrease insulin sensitivity [35], which further exacerbates adipose tissue mobilization and again leads to lipid metabolic disorders [36], thereby triggering a vicious cycle [37]. Insulin is a major regulator of lipogenesis and can influence the growth hormone (GH) signaling pathway by regulating the expression of growth hormone receptor (GHR) [38]. Higher concentrations of GH enhanced the lipolysis of adipose tissue to produce NEFA. However, higher concentrations of GH enhance lipolysis of adipose tissue to produce NEFA. The released NEFA can be metabolized by many tissues for energy production and fat synthesis [39], such as milk fat production in the mammary gland [40], but most NEFA is primarily consumed by the liver [41]. Excessive absorption of NEFA by the liver from plasma leads to the metabolism of ketone bodies in hepatocytes, or the generation of TG from new esterification. Excessive ketone bodies and TG lead to the onset of ketosis and fatty liver syndrome [42] (Figure 10).

Conventionally, cows with high feed efficiency have low RFI (low DMI), and genetic selection in animals with low RFI will improve profitability and optimize the use of food resources. However, with the development of research, more and more scholars suggest that low RFI is more efficient only when the animals are health [43]. The point to the fact that even cows with low RFI would be inefficient if they have a higher propensity to disease [10]. But it also seems to give the study a hint that if we add a selection condition to the classic exploration of disease-related networks or genes, it seems to be a new way forward. In other words, when we look at the genes involved in ketosis in dairy cows, the difference between a sick and a healthy one is one way. But if we consider the relationship between RFI and NEB, it seems that some potential ketosis-related genes can be found in LRFI and HRFI. In fact, we did find candidate factors associated with ketosis in this study (Figure 10). At the same time, the results of this study seem to confirm that our idea is valid.

In the present study, we have theoretical support for the hypothesis that these 2 genes, ACACA/ACC1 [17,44–46] (Acetyl-CoA carboxylase α/I) and ELOVL6 [47] (Elongase of very long chain fatty acids 6), may acts as negative regulators in both low RFI and ketosis. If we trace the relationship between ACACA gene and ketosis in dairy cows, we will find that this study is not the first time to propose that ACACA can be a negative regulator of ketosis. In a meta-analysis, Scares et al. [44] found that ACACA was not only significantly down-regulated in the livers of ketotic cows (compared with healthy cows [17,46,48], but also significantly different in negative energy balance, subclinical ketosis and clinical ketosis. Unfortunately, however, Scares et al. were unable to further explain the reasons for the significant differences in the three dimensions of ACACA, mainly due to the relatively limited data available. From this, we can also see that the basic reference data is of great significance to the construction of multi-dimensional network. Meanwhile, in vitro studies have provided similar evidence. Li et al. [45] found that the mRNA and protein expression of ACAC gradually decreased with the increasing concentration of NEFA in hepatocytes treated with NEFA in vitro.

Acetyl-CoA carboxylase (ACAC/ACC) catalyzes the carboxylation of acetyl-CoA to product malonyl-CoA, a key molecule that controls fatty acid biosynthesis and oxidation in cells [49]. ACACA (ACCα/ACC1, molecular mass 265 kDa) and ACACB (ACCβ/ACC2, molecular mass, 280 kDa) are the only two members (isozymes) of the currently discovered ACACs family, with different physiological roles due to their different subcellular distributions [50]. Malonyl-CoA produced by the carboxylation of cytosolic ACACA is mainly involved in de novo lipogenesis (DNL) [51]. However, the reason for ACACB anchoring to the mitochondrial surface is because of its unique N-terminal domain containing 20 hydrophobic amino acids, so that the production of malonyl-CoA by ACACB carboxylation occurs at the mitochondrial surface [51]. It is well known that malonyl-CoA is a potent endogenous inhibitor of carnitine palmitoyl transferase 1 (CPT-1) [52] located on the mitochondrial surface. Thus, ACACB plays a negative regulation role in the regulation of fatty acid entry into mitochondria and subsequent β-oxidation [53]. Previous studies have mentioned that genetic deletion of both ACACA and ACACB leads to increased hepatic lipid accumulation in vivo [54], but whole-animal deletions of ACACA relative to ACACB is lethal during development [55]. Surprisingly, however, only the liver-specific deletion of only ACACA caused compensation by the ACACB isoform [55]. Compensation by ACACB specifically induces the production of large amounts of malonyl-CoA to maintain physiological levels of malonyl-CoA in the liver lacking ACACA enzyme [55]. The main role of malonyl-CoA at this time is to compensate for the disruption of the hepatic DNL pathway in the liver due to ACACA deficiency. This suggests that, on the one hand, the lack of ACACA does not affect the production of DNL, on the other hand, NEFAs generated by fat mobilization and diminished glycometabolism due to reduced DMI together enhance the β-oxidation capacity of NEFA, which together contribute to increased ketone body production. The pathway in which the ACACA and ELOVL6 are involved and affect ketosis in dairy cows are predicted as shown in Figure 10.

Fatty acids, which are 16 to 18 carbon atoms length, make up the majority of the total fatty acids in cells and are the primary product of DNL in most mammalian tissues [56,57]. This is because the first two-carbon segment of the DNL reaction is acetyl-CoA, and each subsequent round of reaction adds a malonyl-CoA. Since the carboxyl groups added during carboxylation are released as carbon dioxide, the carbonates only promote the reaction and are not involved in the resulting fatty acids. Then, the saturated fatty acids were obtained by reduction, dehydration and re-reduction. After 7 cycles this produces palmitoyl ACP (Acyl carrier protein). Because β-ketoacyl ACP synthetase can only accept 14-carbon acyl group at most, fatty acids synthase (FAS) can only synthesize palmitic acid (Palmitate C16:0) [58]. It can be seen that this series of responses is mainly mediated by ACACA and FAS [47]. Previous studies have shown that more than 90% of endogenous stearates (C18:0) are produced by palmitate (C16:0) involved in the microsomal fatty acid elongation reaction [59]. Palmitate (C16:0) and stearates (C18:0) are subsequently desaturated by SCD (a microsomal Δ9 desaturase) and converted to Palmitoleate (C16:1, n-7) and Oleate (C18:1, n-9/n-7), respectively [47]. ELOVL6, a member of the conserved endoplasmic reticulum localization enzyme family, is a fatty acyl elongation enzyme involved in the formation of long-chain fatty acids [60]. Moon et al found that ELOVL6^-/-^ mice were defective in C16 fatty acid elongation, resulting in a decrease oleate (C18:1, n-9) and accumulation of Palmitate (C16:0) or Palmitoleate (C16:1, n-7) [47]. At the same time, they concluded that deletion of ELOVL6 did not protect mice from developing hepatic steatosis [47].

In this study, we also provided new reference data for RFI biomarkers, namely the SHMT1 gene highly associated with HRFI, STAT3 and IFNAR1 highly associated with LRFI. Similarly, data from previous studies support the conclusions of this study. When enough level of leptin, which signals the body’s energy storage status, are present, it suppresses eating [61]. However, this ability is required with activation of transcription factor STAT3 (signal transducer and activator of transcription 3) [12,62]. Studies of the other two genes we screened, SHMT1 (Serine hydroxy methyl transferase 1) and IFNAR (IFN-α receptor), have focused more on cancer [63,64], and studies on fasting or DMI/RFI need to be explored further. Interestingly, however, Zhang et al found that SHMT inhibits hepatic steatosis, which also seems to indirectly provide information that genes highly associated with HRFI may be negative associated with hepatic lipid deposition and ketosis.

It is also the most regrettable that we did not find the overlapping genes of the four modules in this study (Figure 8a). The absence of overlapping genes across the four modules (Figure 8a) may reflect species-specific adaptations or technical heterogeneity between datasets. Mice (GSE104079) and cows (GSE92398, GSE4304) exhibit divergent regulatory mechanisms for ketosis due to evolutionary differences in lipid metabolism and stress responses. Additionally, variability in experimental designs (e.g., fasting protocols, sequencing platforms) and analytical thresholds may limit direct gene-level comparisons. However, the functional convergence in pathways such as fatty acid metabolism and PPAR signaling (Figure 8b–d) suggests that ketosis is driven by conserved metabolic dysregulation rather than individual gene effects (Figure 10). These findings highlight the need for integrative multi-omics approaches to bridge species and methodological gaps in future studies. But not without harvest, the overlapping gene XPO7 (Exportin 7) emerged as a candidate through WGCNA-RFI and DE-Ket modules (Supplementary Table 2). While direct evidence in dairy cows is limited, its association with fat deposition in yaks [65] and enrichment in fatty acid metabolism pathways (Figure 8b–d; [66]) suggest a conserved role in lipid regulation. XPO7 (Exportin 7) is a nuclear export protein involved in transporting cargo proteins critical for cellular stress responses [67]. Nuclear transport proteins like XPO are critical for shuttling metabolic regulators (e.g., PPAR) that influence hepatic lipid oxidation [37,68]. Although further validation is needed, these indirect links position XPO7 as a plausible mediator of metabolic dysregulation during low feed intake. Future work will explore its functional role in bovine hepatocytes and its association with ketosis phenotypes.

In fact, this study has some limitations and other points of view of the expansion. For example, when we analyzed the overlapping genes of the four modules[DE-ketosis (2,272 genes, |log2FC|>1 & *P* < 0.05), WGCNA-RFI (the hub gene of the turquoise module and the green module, 146 genes), DE-RFI (70 genes, *P* < 0.05) and WGCNA-ADF (the hub gene of the brown module and the yellow module, 269 genes).], we found that there were differences in the number of genes among them. The number of differential genes was the highest in healthy and ketotic cows, which indicated that a large number of pathways were changed after the onset of the disease, which was in line with the metabolic changes of the disease. When we analyzed the association with RFI traits, the number of key genes was 346. The number of genes depends on the definition of the threshold and the selection of the module genes. This number is roughly the same as the number of genes in the WGCNA-ADF module. In fact, what shocked us most was the number of genes and screening criteria of DE-RFI, the minimum number of genes screened and the lowest screening criteria. This result may be due to the treatment diet, which might not have a significant impact or be reflected in the differences in the gene expression in the Holsteins [9]. However, we still use it as a reference for our screening of candidate genes. This may be one of our limitations. Similarly, our research may support a point. Our analysis from a species perspective seems to indicate that individual genes lack commonality in RFI variation. Although this study was carried out on liver tissue, it is consistent with Keogh et al’s findings on muscle (*M. longissimus thoracis et lumborum*) in different species (Charolais and Holstein Friesian) [69].

Previous studies in dairy cows have shown that ACACA expression is downregulated during ketosis and negative energy balance (NEB), correlating with reduced lipogenesis and enhanced fatty acid oxidation [17,46,48]. Soares et al. [44] further confirmed its differential expression across subclinical and clinical ketosis stages, though mechanistic insights remain limited. Our study extends these observations by linking ACACA downregulation specifically to low residual feed intake (RFI), a condition mimicking chronic undernutrition in dairy cows. We demonstrate that ACACA suppression is not merely a consequence of ketosis but may precede metabolic dysregulation during prolonged low DMI (Figure 9a and b). This aligns with in vitro evidence showing that elevated NEFA levels directly inhibit ACACA expression [45], suggesting a feed-forward loop exacerbating lipid mobilization and ketogenesis. In ruminants, a recent transcriptome sequencing study of adipose tissue inflammation, remodeling, and lipid metabolism in perinatal dairy cows suggested that ELOVL6 is an upstream inhibitor of lipid synthesis [70]. This study highlights its role in adipose lipid remodeling, though its hepatic function in ketosis remained unexplored. We provide the first evidence that ELOVL6 is downregulated in the liver of ketotic cows with low RFI (Figure 9a and b). This contrasts with adipose-specific suppression reported by Salcedo-Tacuma et al. [70],, suggesting tissue-specific regulatory mechanisms during metabolic stress. In the liver, reduced ELOVL6 activity may impair the elongation of palmitate (C16:0) to stearate (C18:0), limiting substrate availability for Δ9-desaturase (SCD) and disrupting the balance between saturated and unsaturated fatty acids. This imbalance could exacerbate endoplasmic reticulum stress, a known trigger of hepatic lipidosis [71]. Our findings position ELOVL6 as a dual regulator of lipid metabolism in both adipose and hepatic tissues, with distinct roles in ketosis pathogenesis. XPO7 mediates nuclear export of proteins and miRNAs, with emerging roles in lipid metabolism. Ji et al. (2020) identified XPO7 as a miRNA target in yak adipose tissue, suggesting its involvement in fat deposition. However, no prior studies have linked XPO7 to ketosis in dairy cows. Our study is the first to implicate XPO7 in ketosis through its co-expression with lipid metabolism genes (e.g., HADHB) in RFI/fasting modules (Figure 8b). While direct evidence in ruminants is sparse, XPO’s interaction with transcriptional regulators like PPAR [68] suggests it may modulate nuclear-cytoplasmic shuttling of metabolic sensors during energy stress. This positions XPO7 as a novel candidate for further exploration in bovine hepatic adaptation to low feed intake.

Our identification of ACACA, ELOVL6, and XPO7 as key regulators of ketosis under low RFI conditions adds new dimensions to existing knowledge. While ACACA’s role in ketosis has been documented [17,46,48], our data uniquely associate its suppression with chronic low DMI, highlighting its potential as an early biomarker. Similarly, ELOVL6’s downregulation in the liver of ketotic cows contrasts with its adipose-specific suppression during periparturient inflammation [70], suggesting tissue-specific adaptations in lipid metabolism. The novel inclusion of XPO7, previously linked to fat deposition in yaks (Ji et al., 2020), underscores the importance of nuclear transport mechanisms in metabolic stress. These findings collectively advance our understanding of ketosis pathogenesis and open avenues for targeted interventions. While this study identifies potential regulators of ketosis induced by low feed intake through cross-species integration of bovine and murine data, the limitations of such analyses warrant careful interpretation. First, core metabolic pathways (e.g., mitochondrial fatty acid synthesis pathway) exhibit profound evolutionary conservation [72]. For example, ELOVL6, a key lipid metabolism regulator, belongs to a highly conserved endoplasmic reticulum-localized enzyme family involved in long-chain fatty acid biosynthesis [73], supporting the rationale for cross-species candidate gene prioritization. However, interspecies anatomical and physiological divergences may introduce biases in direct comparisons of gene expression patterns. Our strategy focuses on conserved pathway hubs rather than species-specific phenotypic variations, thereby optimizing target discovery efficiency. Future studies should validate these candidates using bovine primary hepatocyte models to delineate species-specific regulatory mechanisms.

## Conclusion

In this study, the key genes of ketosis caused by low feed intake were analyzed, and it was found that ACACA, ELOVL6 and XPO7 genes could be used as regulatory factors affecting ketosis. At the same time, we also provided three genes (HRFI, STAT3 and IFNAR1) for further consideration to be added as RFI biomarkers. These genes can expand the gene pool related of ketosis and RFI in dairy cows.

## Compliance and ethics

The authors declare that they have no conflicts of interest.

## Supplementary Material

Supplementary Table1.xlsx

Supplementary Figure 2.tif

Supplementary Figure 1.tif

Supplementary Figure 3.tif

Supplementary Table4.xlsx

Supplementary Table3.xlsx

Supplementary Table2 .xlsx

Supplementary Figure 4.tif

## Data Availability

The original contributions presented in the study are included in the article/Supplementary Material, further inquiries can be directed to the corresponding author/s.
