# Supplementary figures and images for: Identification of key factors causing ketosis in dairy cows with low feed intake
Source: Anim Biotechnol. 2025 Apr 4;36(1):2487089. doi: 10.1080/10495398.2025.2487089 (PMC12674265; doi:10.1080/10495398.2025.2487089)

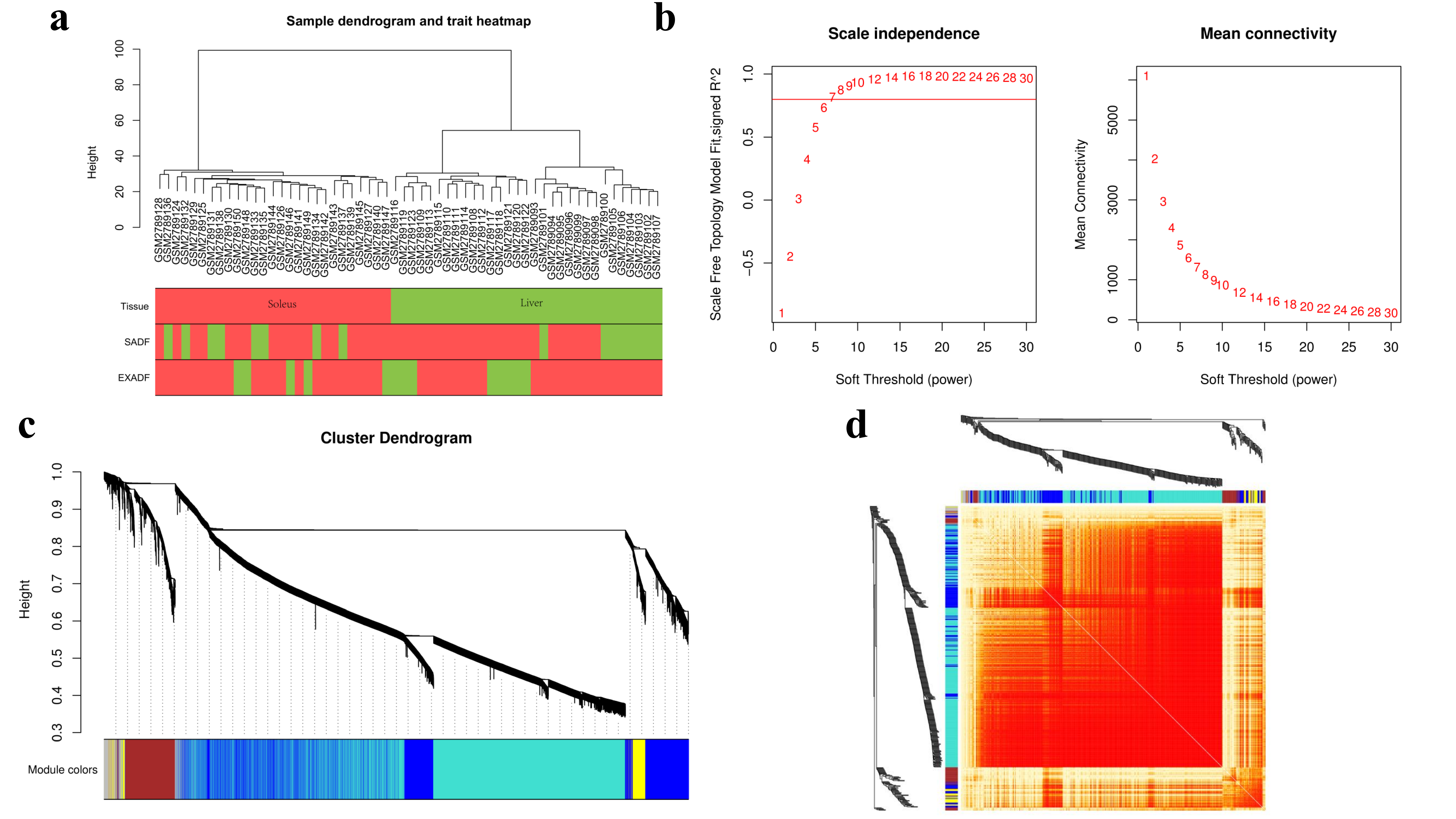

Supplement: Supplementary Figure 2.tif [file LABT_A_2487089_SM9466.tif]

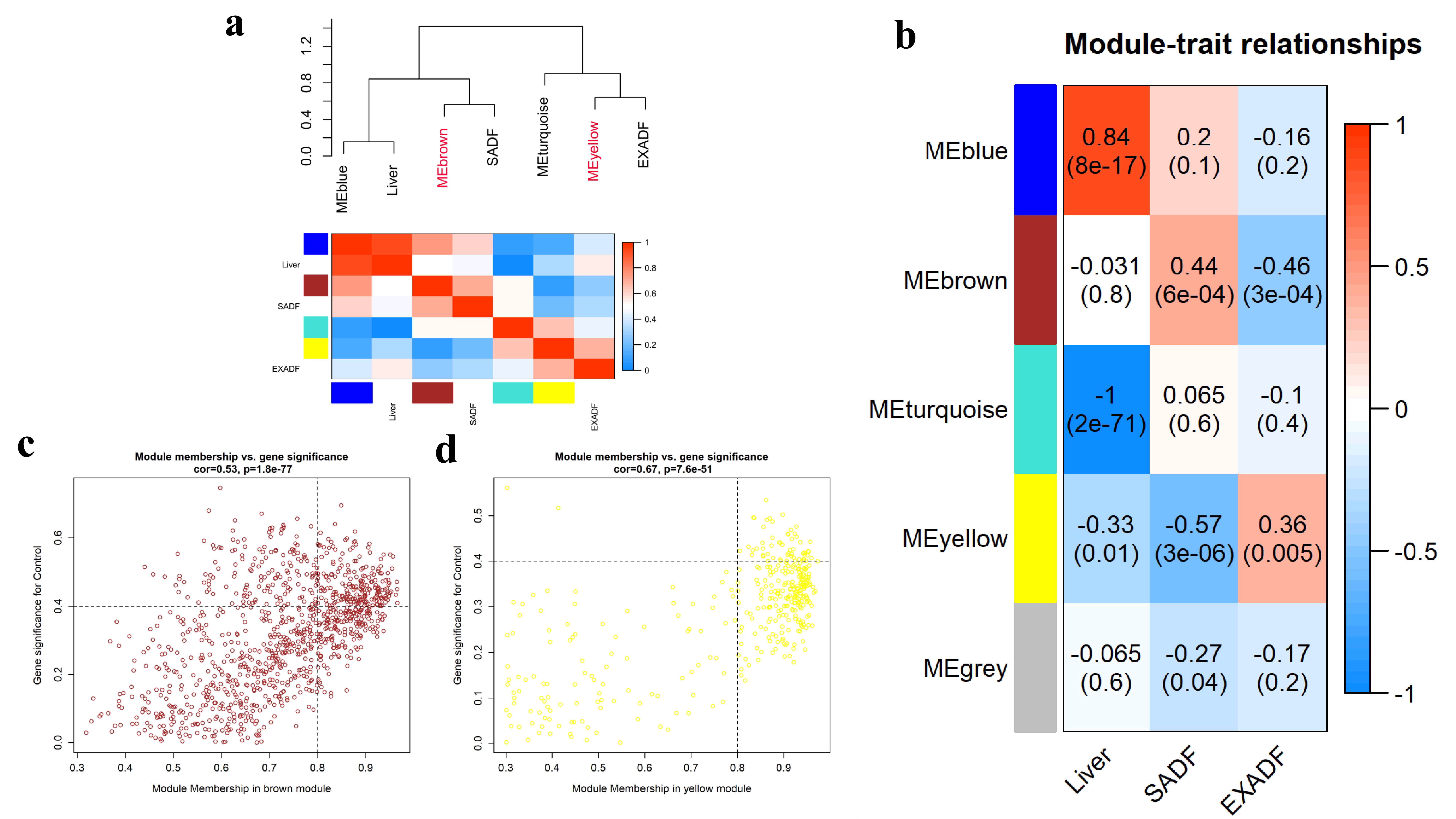

Supplement: Supplementary Figure 3.tif [file LABT_A_2487089_SM9464.tif]

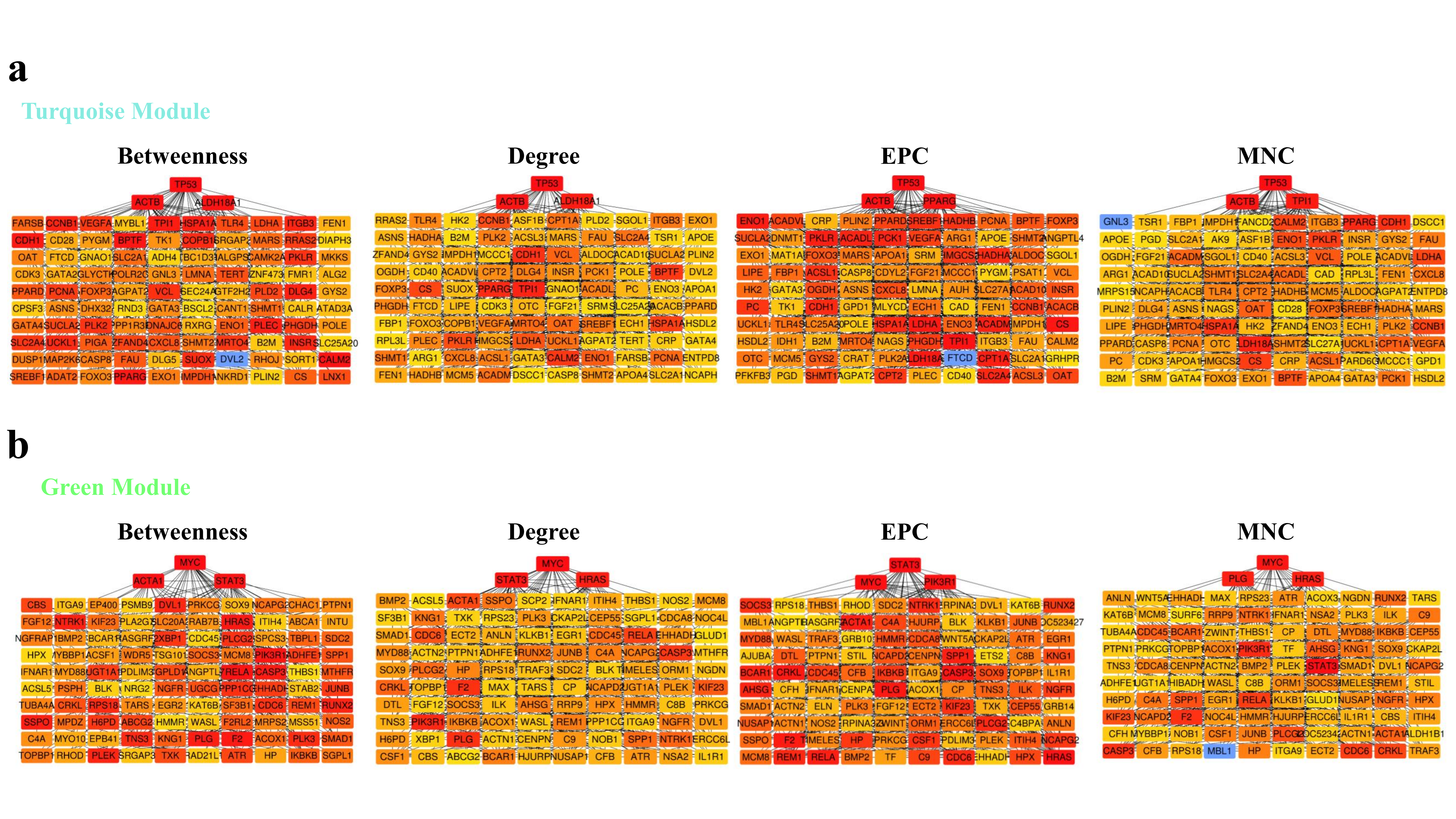

Supplement: Supplementary Figure 4.tif [file LABT_A_2487089_SM9460.tif]
